# Association between vitamin K intake and depressive symptoms in US adults: Data from the National Health and Nutrition Examination Survey (NHANES) 2013–2018

**DOI:** 10.3389/fnut.2023.1102109

**Published:** 2023-03-22

**Authors:** Yuyi Zhang, Weiliang Tan, Xiaolan Xi, Hui Yang, Ke Zhang, Shengnan Li, Xuefen Chen, Hui Zuo

**Affiliations:** ^1^School of Public Health, Suzhou Medical College of Soochow University, Suzhou, China; ^2^Suzhou Medical Association, Suzhou, China; ^3^School of Public Health, Shanghai University of Traditional Chinese Medicine, Shanghai, China; ^4^Jiangsu Key Laboratory of Preventive and Translational Medicine for Geriatric Diseases, Suzhou Medical College of Soochow University, Suzhou, China

**Keywords:** depression, menaquinone-4, cross-sectional study, National Health and Nutrition Examination Survey, vitamin K intake

## Abstract

**Background:**

The relationship between vitamin intake and depression has attracted increasing attention. However, several studies examining such relationship among populations at different age groups have produced inconsistent findings. This study was aimed to investigate the cross-sectional association between vitamin K intake and depressive symptoms in US adults.

**Methods:**

We used the data from a nationally representative sample of 11,687 adults from the 2013 to 2018 National Health and Nutrition Examination Survey (NHANES). Vitamin K intake was assessed by the 24-h dietary recall at the first day. Depressive symptoms were assessed using the 9-item Patient Health Questionnaire (PHQ-9). Logistic regression and generalized additive model were used to examine the association between vitamin K intake and depressive symptoms.

**Results:**

The weighted prevalence of depressive symptoms was 10.2% (8.0% in men and 12.0% in women). We observed a significant inverse linear relationship between vitamin K intake and depressive symptoms in models adjusted for age, sex, race/ethnicity, marital status, educational status, family poverty income ratio (PIR), home status, body mass index (BMI), smoking status, physical activity, sleep disorders, hypertension, hyperlipidemia, and diabetes. The odds ratios (OR) (95% CI) for the highest compared with the lowest quartile of vitamin K intake was 0.68 (95% CI: 0.52, 0.89, *p*-trend < 0.05). The association was similar in subgroups stratified by age, sex, race/ethnicity, marital status, educational status, PIR, home status, BMI, smoking status, physical activity, sleep disorders, hypertension, hyperlipidemia, and diabetes.

**Conclusion:**

Vitamin K intake was inversely and independently associated with the odds of depressive symptoms in the US adults. Prospective studies are warranted to confirm our findings.

## Introduction

Depression is a major public health problem and the second most serious disease in the world after cardiovascular diseases with persistence and recurrence characteristics (1). It is predicted that depression will become the primary cause of the global burden of disease by 2030 (2). The reported percentage of depression was 3.8% globally and 5.0% among adults as of 2021 (3). Depression is the most prevalent psychiatric disorder in the US (4). A study based on data from the National Health and Nutrition Examination Survey (NHANES) in 2017–2018 reported that 8.7% of the US adults suffered from the condition (5). Depression affects individual’s quality of life (6) and body functions, such as muscle strength or motor function (7, 8). Moreover, it can cause or aggravate physical diseases including cardiovascular diseases (9), diabetes (10), and even suicide (11). Although depression is a common disease, its pathogenesis remains unclear (12).

Accumulating studies have linked nutrition to the risk of depression, especially vitamins. For example, vitamin D (13) and vitamin B_12_ deficiency (14) were suggested as risk factors for depression. Vitamin E can protect against nerve damage, and its low intake has been linked to depressed mood (15). Other micronutrients including zinc, magnesium and selenium were also associated with depression by previous reports (16). However, few studies reported the relationship between vitamin K and depressive symptoms.

Vitamin K comes in two biologically active forms: phylloquinone (vitamin K1) and menaquinone (vitamin K2) (17). In the past decades, more attention in vitamin K has been paid on blood coagulation function (17), cardiovascular and bone health (18). Vitamin K has also been suggested to play an important role in the brain (19). High concentration of sphingolipids was found in both neuronal and glial cell membranes. Vitamin K can modulate the activity of key enzymes in the sphingolipid biosynthetic pathway, thereby affecting their synthesis and metabolism (20). Gas6, a member of the vitamin K-dependent proteins, plays a key role in cellular homeostasis through its cell-signaling action in neurons and glial cells (21). In particular, it has been linked to protection against oxidative stress in the brain, a mechanism thought to be important in influencing depression (19). In addition, vitamin K concentrations in the brain in animal models have been observed in parallel with its dietary intake (22). Vitamin K is present predominantly as menaquinone-4 (MK-4) in the brain. It was recently confirmed that MK-4 makes up 98% of the total vitamin K in the brains of 6-month-old and 21-month-old rats (23). MK-4 is unique among the menaquinones since it is not synthesized by bacteria (24).

A Japanese study reported a relation between low vitamin K intake and depressive symptoms among people aged 65 years and older (25). Similar results were seen in a sample of US with an average age of 61.3 years (26). However, the two studies included only older participants and the sample sizes were relatively small. Moreover, an inconsistent association was observed in Spanish children (27). Thus, the association of vitamin K intake with depressive symptoms needs to be further investigated. To the best of our knowledge, there have been no studies on vitamin K and depressive symptoms in adults at all age groups. Therefore, the aim of the present study was to investigate the association between vitamin K intake and depressive symptoms in adults, including young adults, middle-aged, and older people, using NHANES data 2013–2018.

## Materials and methods

### Study population

**FIGURE 1:**
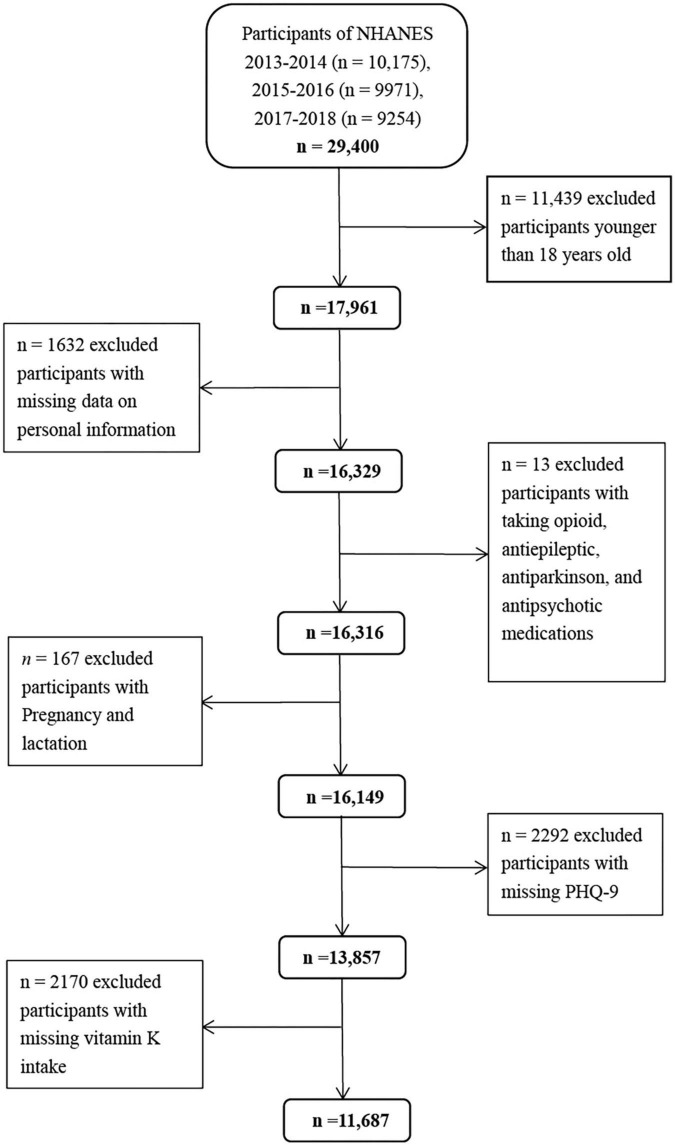
Flow chart of study participants.

The NHANES program has been conducted as a series of cross-sectional surveys by the Centers for Disease Control and Prevention. It uses a complex, stratified multistage, probability sampling design to select a nationally representative sample of non-institutionalized US population every 2 years (28, 29). Individuals selected are invited to participate in an interview at home and a health examination at mobile examination centers (MEC) to evaluate their health and nutritional status (30). In the present study, we used the data from the three waves of the 2013–2018 NHANES. There was a total of 17,961 adults aged ≥ 18 years. We excluded individuals with missing data on personal information (*n* = 1632), taking opioid, antiepileptic, antiparkinson, and antipsychotic medications (*n* = 13), pregnancy and lactation (*n* = 167), missing PHQ-9 (*n* = 2292), and vitamin K intake (*n* = 2170) (Figure 1). Correspondingly, a total of 11,687 participants (5,433 men and 6,254 women) were included in the final analyses. Compared with the lost participants, those retained were generally younger (46.6 vs. 47.8 years, *p* < 0.05). No differences in mean BMI and vitamin K intake were found (*p* > 0.05). The National Center for Health Statistics Ethics Review Board approved the NHANES, and all participants provided signed informed consent.

### Definition of depressive symptoms

Depressive symptoms were evaluated using 9-item Patient Health Questionnaire (PHQ-9) in 2013–2018. The questions were asked at the MEC by trained interviewers. This screening instrument mainly measured the frequency of self-reported depression-related symptoms in the preceding 2 weeks (31). Each item on PHQ-9 was scored from 0 (not at all) to 3 (nearly every day). The corresponding total scores ranged from 0 and 27 (32). In this study, those with PHQ-9 ≥ 10 were defined having depressive symptoms (33).

### Assessment of vitamin K intake

Data on vitamin K intake, from both diet and dietary supplements, were obtained from the 24-h dietary recall during the survey period (2013–2018) (34). All participants were eligible for two 24-h dietary recall interviews. The first dietary recall interview was conducted in-person in the MEC and the second interview by telephone 3 to 10 days later. Upon completion of the in-person interview, participants were given measuring cups, spoons, a ruler, and a food model booklet, which contained two-dimensional drawings of the various measuring guides available in the MEC, to use for reporting food amounts during the telephone interview. What is noteworthy is that these participants did not receive uniform training in food measurement. Therefore, we only used the data from the first day considering the accuracy of the 24-h dietary recall data (34, 35). During the interview, participants were asked to recall all foods and beverages consumed from midnight to midnight of the past 24 h. Vitamin K intake from each food or beverage was calculated using the US Department of Agriculture’s Food and Nutrient Database for Dietary Studies (FNDDS)^1^.

### Covariates

During the NHANES 2013–2018, a home interview section collected information about socio-demographic, lifestyle, and health-related factors. The body measures data were collected in the MEC by trained health technicians. In this study, we considered covariates including socio-demographic [sex, age, education status, ethnicity, marital status, body mass index (BMI), and family poverty income ratio (PIR)], lifestyle (home status, smoking status, and physical activity), health-related factors (self-report sleep disorders, hypertension, hyperlipidemia, and diabetes).

### Statistical analyses

Categorical variables were reported as numbers and percentages. Differences in socio-demographic, lifestyle, and health-related factors between the participants with and without depressive symptoms were compared using χ^2^ tests. Vitamin K intake was categorized into quartiles. Log transformation was applied to normalize the distribution of vitamin K intake. We used logistic regression to evaluate the association between vitamin K intake and depressive symptoms. Corresponding odds ratios (ORs) and 95% CIs were reported for the quartiles and per 1-SD increment of vitamin K intake, respectively.

To estimate the independent association of vitamin K intake (by quartiles and as a continuous variable) with depressive symptoms, we constructed three multivariable models to adjust for potential covariates in addition to the unadjusted model: Model 1 was the model adjusted for age (18–44, 45–59, or ≥ 60 years), and sex; Model 2 was further adjusted for race/ethnicity (Mexican American, other Hispanic, non-Hispanic White, non-Hispanic Black, or other Race-Including Multi-racial), marital status (married/partner, widowed/divorced, or never married), educational status (<high school, high school/GED, or >high school), PIR (<1 or ≥1), home status (owned or being bought, rented, or other arrangement), BMI (<25, 25–29.9, or ≥30 kg/m^2^), smoking status (every day, some days, or not at all), and physical activity (no and low or moderate and high); Model 3 was adjusted as for model 2 plus sleep disorders (yes or no), hypertension (yes or no), hyperlipidemia (yes or no), and diabetes (yes or no). In addition, tests for trend (*p*-value for trend) were performed by entering the quartiles of vitamin K intake as a continuous variable and rerunning the corresponding regression models. Generalized additive model was used to examine potential non-linear association of vitamin K intake levels with depressive symptoms.

Potential effect modification by sex, age, race/ethnicity, marital status, educational status, PIR, home status, BMI, smoking status, physical activity, sleep disorders, hypertension, hyperlipidemia, and diabetes was assessed in stratified analyses. For each stratification variable separately, we estimated the significance of interactions based on first-degree multiplicative models. SAS (version 9.4; SAS Institute, Inc., Cary, NC, USA) and R (version 4.5.0^2^ ; The R Foundation for Statistical Computing, Vienna, Austria) were used for all statistical analyses. All tests were two-sided, and a *P* value less than 0.05 was considered statistically significant. We use weighted analysis to consider the complex sampling design of NHANES. Details are as follows: https://www.cdc.gov/nchs/nhanes/index.htm.

## Results

### Characteristics of the participants

Among the 11,687 participants, 1,298 (weighted prevalence: 10.2%) were defined with depressive symptoms. The prevalence was higher in women (12.0%) than that in men (8.0%) (*p* < 0.001). Characteristics of the participants by depressive symptoms are shown in Table 1. Statistically significant differences were observed between the participants with and without depressive symptoms regarding socio-demographic, lifestyle and health-related factors. Compared with the participants without depressive symptoms, those with depressive symptoms were more likely to be women and widowed/divorced, and have less education, and lower PIR, and smoke every day, live with a rented home, and have obesity, no and low physical activity, sleep disorders, hypertension, hyperlipidemia, diabetes (*p* < 0.05). Moreover, the levels of the vitamin K intake were significantly lower in the participants with depressive symptoms (Table 1). The median vitamin K intake was 128.7 mcg/d (interquartile range: 122.6–134.8 mcg/d) among the participants with depressive symptoms and 98.1 mcg/d (interquartile range: 90.3–105.9 mcg/d) in their counterparts.

**TABLE 1:** Characteristics of the participants by depressive symptoms (*n* = 11,687).

Variable	Without depressive symptoms (*n* = 10,386)	Depressive symptoms (*n* = 1,301)	*p*
Sex			<0.001
Men	4,949 (47.2)	484 (36.5)	
Women	5,437 (52.8)	817 (63.5)	
Age (years)			0.835
18–44	4,787 (46.8)	512 (45.6)	
45–59	2,392 (26.9)	346 (27.9)	
≥60	3,207 (26.3)	443 (26.5)	
Race/ethnicity			0.128
Mexican American	1,538 (8.8)	189 (7.9)	
Other Hispanic	1,035 (5.8)	167 (7.3)	
Non-Hispanic White	4,124 (66.5)	538 (63.9)	
Non-Hispanic Black	2,175 (10.7)	269 (11.7)	
Other Race-Including Multi-racial	1,514 (8.2)	138 (9.2)	
Marital status			<0.001
Married/partner	5,817 (63.7)	550 (48.0)	
Widowed/divorced	2,104 (17.8)	434 (30.5)	
Never married	1,874 (18.5)	258 (21.5)	
Education status			<0.001
<High school	2,029 (12.4)	399 (21.0)	
High school/GED	2,474 (23.5)	338 (28.3)	
>High school	5,876 (64.1)	561 (50.7)	
PIR			<0.001
<1	2,861 (13.7)	532 (26.4)	
≥1	7,525 (86.3)	769 (73.6)	
Home status			<0.001
Owned or being bought	6,138 (67.4)	621 (55.2)	
Rented	3,770 (30.5)	592 (41.4)	
Other arrangement	249 (2.1)	48 (3.4)	
BMI (kg/m^2^)			<0.001
<25	2,981 (28.3)	302 (25.9)	
25–29.9	3,240 (31.8)	318 (23.1)	
≥30	4,078 (39.9)	665 (51.0)	
Smoking status			<0.001
Every day	1,880 (27.4)	505 (55.7)	
Some days	3,777 (50.7)	256 (23.7)	
Not at all	1,347 (21.9)	194 (20.6)	
Physical activity			<0.001
No and low	3,895 (34.1)	611 (43.4)	
Moderate and high	6,491 (65.9)	684 (56.6)	
Sleep disorders			<0.001
Yes	2,185 (23.5)	657 (53.7)	
No	8,194 (76.5)	641 (46.3)	
Hypertension			<0.001
Yes	3,659 (32.5)	637 (44.8)	
No	6,721 (67.5)	661 (55.2)	
Hyperlipidemia			0.004
Yes	3,540 (34.1)	545 (40.0)	
No	6,791 (65.9)	738 (60.0)	
Diabetes			<0.001
Yes	1,376 (10.4)	272 (17.1)	
No	8,729 (89.6)	986 (82.9)	
Vitamin K intake (mcg/d)			<0.001
Quartile 1 (<40.2)	2,475 (21.6)	441 (32.3)	
Quartile 2 (40.2–74.1)	2,585 (24.3)	339 (25.5)	
Quartile 3 (74.2–133.2)	2,646 (26.5)	278 (22.4)	
Quartile 4 (≥133.3)	2,680 (27.6)	243 (19.8)	

All variables are expressed as numbers and weighted percentages (%). *P* values were calculated using χ^2^ tests. BMI, body mass index; GED, General Educational Development; PIR, ratio of family income to poverty.

### Vitamin K intake and depressive symptoms

**FIGURE 2:**
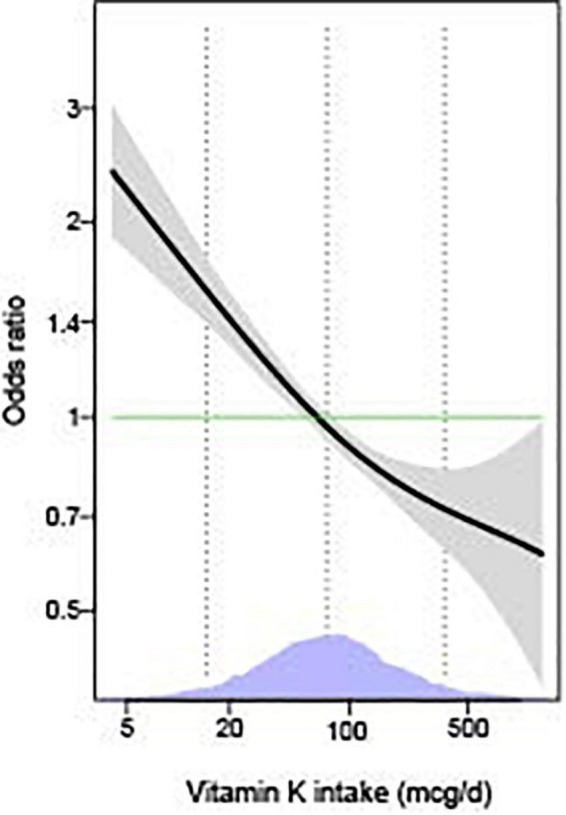
The associations of vitamin K with depressive symptoms by generalized additive models (*n* = 11,687). The models were adjusted for age (18–44, 45–59, or ≥60 years), sex (men or women), race/ethnicity (Mexican American, other Hispanic, non-Hispanic White, non-Hispanic Black, or other Race-Including Multi-racial), marital status (married/partner, widowed/divorced, or never married), educational status (<high school, high school/GED, or >high school), PIR (<1 or ≥1), home status (owned or being bought, rented, or other arrangement), BMI (<25, 25–29.9, or ≥30 kg/m^2^), smoking status (every day, some days, or not at all), physical activity (no and low or moderate and high), sleep disorders (yes or no), hypertension (yes or no), hyperlipidemia (yes or no), and diabetes (yes or no). The solid lines show OR and the shaded areas 95% CI. The dashed lines show OR by linear regression on logarithmic scale. Density plots indicate the distributions of log-transformed vitamin K intake, and dotted lines denote the 10, 50, and 90^th^ percentiles. BMI, body mass index; GED, General Educational Development; PIR, ratio of family income to poverty.

As shown in Table 2, we observed that higher levels of vitamin K intake were associated with decreased odds of depressive symptoms in the unadjusted model. The association remained significant in the models adjusted for age and sex, models with further adjustments for race/ethnicity, marital status, educational status, PIR, home status, BMI, smoking status, physical activity, and in the fully adjusted models with additional adjustments for sleep disorders, hypertension, hyperlipidemia, and diabetes (all *p* < 0.05). The multivariable-adjusted OR for depressive symptoms was 0.68 (95% CI: 0.52, 0.89) for the highest compared with the lowest quartile of vitamin K, and 0.84 (95% CI: 0.75, 0.94) per 1-SD increment of log-transformed vitamin K intake. We obtained similar results based on Model 3 after further adjustment for B vitamins, zinc, magnesium, selenium, and other micronutrients (data not shown). Generalized additive model showed that the association between vitamin K intake and depressive symptoms was essentially linear across the distribution of vitamin K intakes (Figure 2). In addition, similar results were found in the associations of vitamin K from diet and dietary supplements separately with depressive symptoms (data not shown).

**TABLE 2:** Odds ratios (95% confidence intervals) for depressive symptoms by vitamin K intake^a^ (*n* = 11,687).

Vitamin K intake (mcg/d)	Case/*n*	Crude	Model 1^b^	Model 2^c^	Model 3^d^
Quartile 1	441/2902	1.00 (ref.)	1.00 (ref.)	1.00 (ref.)	1.00 (ref.)
Quartile 2	339/2934	0.70 (0.58, 0.85)	0.71 (0.59, 0.86)	0.84 (0.64, 1.09)	0.84 (0.61, 1.14)
Quartile 3	278/2024	0.57 (0.45, 0.72)	0.59 (0.46, 0.75)	0.76 (0.60, 0.95)	0.74 (0.58, 0.95)
Quartile 4	243/2927	0.48 (0.40, 0.58)	0.49 (0.41, 0.59)	0.66 (0.51, 0.87)	0.68 (0.52, 0.89)
*p*-trend		<0.001	<0.001	0.013	0.015
Continuous	1301/11687	0.73 (0.68, 0.79)	0.74 (0.68, 0.80)	0.83 (0.75, 0.92)	0.84 (0.75, 0.94)
*p*		<0.001	<0.001	<0.001	0.003

^a^Logistic regression models were used to calculate the odds ratios and 95% confidence intervals.

^b^Model 1 was adjusted for age (18–44, 45–59, or ≥ 60 years) and sex.

^c^Model 2 was adjusted as for model 1 plus race/ethnicity (Mexican American, other Hispanic, non-Hispanic White, non-Hispanic Black, or other Race-Including Multi-racial), marital status (married/partner, widowed/divorced, or never married), educational status (<high school, high school/GED, or >high school), PIR (<1 or ≥1), home status (owned or being bought, rented, or other arrangement), BMI (<25, 25–29.9, or ≥30 kg/m^2^), smoking status (every day, some days, or not at all), and physical activity (no and low or moderate and high).

^d^Model 3 was adjusted as for model 2 plus sleep disorders (yes or no), hypertension (yes or no), hyperlipidemia (yes or no), and diabetes (yes or no).

### Stratified analyses

**FIGURE 3:**
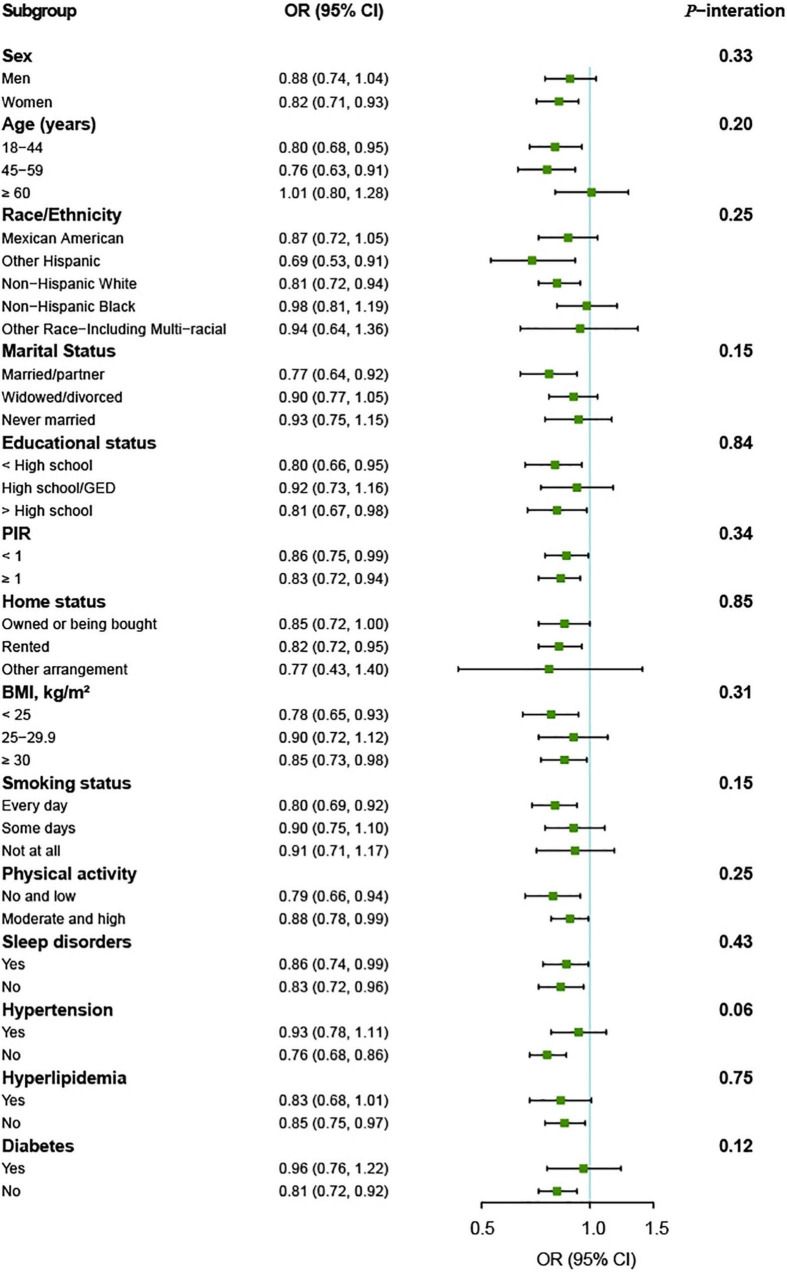
Vitamin K intake and depressive symptoms by different characteristics of the participants (*n* = 11,687). OR (95% CI) are reported per 1-SD increment of log-transformed vitamin K intake. The models were adjusted for age (18–44, 45–59, or ≥60 years), sex (men or women), race/ethnicity (Mexican American, other Hispanic, non-Hispanic White, non-Hispanic Black, or other Race-Including Multi-racial), marital status (married/partner, widowed/divorced, or never married), educational status (<high school, high school/GED, or >high school), PIR (<1 or ≥1), home status (owned or being bought, rented, or other arrangement), BMI (<25, 25–29.9, or ≥30 kg/m^2^), smoking status (every day, some days, or not at all), physical activity (no and low or moderate and high), sleep disorders (yes or no), hypertension (yes or no), hyperlipidemia (yes or no), and diabetes (yes or no). BMI, body mass index; GED, General Educational Development; PIR, ratio of family income to poverty.

The association between vitamin K intake and the risk of depressive symptoms was similar in subgroups stratified by age, sex, race/ethnicity, marital status, educational status, PIR, home status, BMI, smoking status, physical activity, sleep disorders, hypertension, hyperlipidemia, and diabetes. There were no significant interactions for vitamin K intake and these variables (all *p* for interaction > 0.05) (Figure 3).

## Discussion

In this large cross-sectional study of the 11,687 nationally representative US adults, the weighted prevalence of depressive symptoms was 10.2%. It required that the state should pay more attention to mental health of the adults, especially women, the widowed/divorced, those with less education, and lower PIR, living with rented home, obesity, smoking every day, no and low physical activity, sleep disorders, hypertension, hyperlipidemia, diabetes, which is in agreement with previous studies (36, 37). We observed that a higher vitamin K intake was strongly associated with lower odds of depressive symptoms, which was consistent with our hypothesis. Unlike the two previous studies (25, 26), however, the present study also included population aged 18 to 45 years and therefore had better generalizability to different age groups. The inverse association between vitamin K intake and depressive symptoms was independent of potential confounding factors, and was similar across subgroups stratified by age, sex, race/ethnicity, marital status, educational status, PIR, home status, BMI, smoking status, physical activity, sleep disorders, hypertension, hyperlipidemia, and diabetes.

The inverse association of vitamin K intake with depressive symptoms was consistent with previous epidemiological studies from US and Japan (25, 26). An animal study observed that vitamin K deficiency due to dietary depletion or by warfarin treatment was associated with hypo-activity and a lack of exploratory behavior in rats (38). In addition, Turker et al. found a significantly higher frequency of depression during the treatment of patients with atrial fibrillation using warfarin, a vitamin K antagonist (39), which can be indirectly supported by our results. Notably, the likelihood of depressive symptoms decreased continuously across the entire distribution of vitamin K intake levels. Participants in the highest quartile of vitamin K intake had nearly half odds of depressive symptoms compared with those in the lowest quartile. The associations between vitamin K intake and depressive symptoms were essentially unaltered in the three models with gradual adjustments for potential confounding factors, and did not differ between the multiple subgroups. This indicates that the association between vitamin K intake and depressive symptoms is unlikely to be mediated through traditional risk factors for depressive symptoms, such as sleep disorders, obesity, and diabetes.

The exact mechanism of vitamin K in association with depression has not yet been fully clarified. A previous meta-analysis showed increased levels of oxidative stress in people with depression (40). The study from Ferland et al. has shown that lifetime low-vitamin K diet was associated with higher levels of ceramides in the hippocampus in rats (41), whereas increased concentrations of ceramide have been associated with proinflammatory processes, production of reactive oxygen species, and inhibition of neuronal survival. Lack of neurogenesis in the hippocampus has been proposed as a possible cause of major depression (41). In view of this, we speculate that increasing vitamin K intake can increase neurogenesis in the hippocampus and reduce oxidative stress in the brain, and consequently reduce depressive symptoms.

Guylaine had pointed out that MK-4 protects against oxidative stress (23). Hence, we further speculate that the effect of vitamin K on depressive symptoms might be due to the action of MK-4.

To the best of our knowledge, our study is the first to examine the association between vitamin K intake and depressive symptoms in the entire adult population, from young adults to the oldest old. Second, the study population came from NHANES study with nationally representative samples, which provided enough power for the findings. Third, we considered a wide range of potential confounders to better estimate the association between vitamin K intake and depressive symptoms. However, there are also several limitations worth mentioning. First, due to the cross-sectional design of this study, we are unable to explore any causal relationships between vitamin K intake and depressive symptoms. Second, we only used the dietary recall data at the first day in this study. Random measurement errors were inevitable and may have biased the findings. Third, the findings from the US may not be generalizable to other populations due to differences in genetic background, metabolism, and vitamin K intake levels.

## Conclusion

In the present study, we observed a strong and inverse association between vitamin K intake and depressive symptoms among US adults. It may suggest a potential role for vitamin K in the preventing and treating depression. However, prospective studies are needed to elucidate the causality between increased vitamin K intake and risk of depression.

## Data availability statement

Publicly available datasets were analyzed in this study. This data can be found here: https://www.cdc.gov/nchs/nhanes/index.htm.

## Ethics statement

The studies involving human participants were reviewed and approved by the National Center for Health Statistics Ethics Review Board. The patients/participants provided their written informed consent to participate in this study. Written informed consent was obtained from the individual(s) for the publication of any potentially identifiable images or data included in this article.

## Author contributions

YZ and HZ designed this research. YZ conducted the statistical analyses. YZ, XX, HY, KZ, and SL wrote the manuscript. WT, XC, and HZ gave a critical revision of the final version. All authors have made substantial contributions to the work, read the manuscript, agreed the submission of this work to the journal, and accept responsibility for the manuscript’s contents.

## Footnotes

1
http://www.ars.usda.gov/ba/bhnrc/fsrg

2
www.r-project.org
